# Hosts mobility and spatial spread of *Rickettsia rickettsii*

**DOI:** 10.1371/journal.pcbi.1006636

**Published:** 2018-12-26

**Authors:** Gina Polo, Carlos Mera Acosta, Marcelo B. Labruna, Fernando Ferreira, Dirk Brockmann

**Affiliations:** 1 Laboratory of Epidemiology and Biostatistics. Department of Preventive Veterinary Medicine and Animal Health. University of São Paulo, São Paulo, Brazil; 2 Robert Koch-Institute, Berlin, Germany; 3 Physics Institute, University of São Paulo, São Paulo, Brazil; 4 Laboratory of Parasitic Diseases. Department of Preventive Veterinary Medicine and Animal Health. University of São Paulo, São Paulo, Brazil; 5 Institute for Theoretical Biology and Integrative Research Institute for the Life Sciences, Humboldt Universität zu Berlin, Berlin, Germany; University of California, Los Angeles, UNITED STATES

## Abstract

There are a huge number of pathogens with multi-component transmission cycles, involving amplifier hosts, vectors or complex pathogen life cycles. These complex systems present challenges in terms of modeling and policy development. A lethal tick-borne infectious disease, the Brazilian Spotted Fever (BSF), is a relevant example of that. The current increase of human cases of BSF has been associated with the presence and expansion of the capybara *Hydrochoerus hydrochaeris*, amplifier host for the agent *Rickettsia rickettsii* and primary host for the tick vector *Amblyomma sculptum*. We introduce a stochastic dynamical model that captures the spatial distribution of capybaras and ticks to gain a better understanding of the spatial spread of the *R. rickettsii* and potentially predict future epidemic outcomes. We implemented a reaction-diffusion process in which individuals were divided into classes denoting their state with respect to the disease. The model considered bidirectional movements between base and destination locations limited by the carrying capacity of the environment. We performed systematic stochastic simulations and numerical analysis of the model and investigate the impact of potential interventions to mitigate the spatial spread of the disease. The mobility of capybaras and their attached ticks was significantly influenced by the birth rate of capybaras and therefore, disease propagation velocity was higher in places with higher carrying capacity. Some geographical barriers, generated for example by riparian reforesting, can impede the spatial spread of BSF. The results of this work will allow the formulation of public actions focused on the prevention of BSF human cases.

## Author summary

Complex systems as the Brazilian Spotted Fever (BSF), present challenges in terms of modeling and policy development. BSF human cases have been associated with the presence and expansion of the capybara *Hydrochoerus hydrochaeris*, amplifier host for the agent *Rickettsia rickettsii* and primary host for the tick vector *Amblyomma sculptum*. We developed a reaction-diffusion system for the spread of BSF by considering the spatial structure and migration of amplifier hosts to gain a better understanding of the spatial spread of the *R. rickettsii* and potentially predict future epidemic outcomes. We performed stochastic simulations and numerical analysis to investigate the impact of potential interventions to mitigate the spatial spread of the disease. Our results indicate that as we vary the amount of capybaras’ food sources, the velocity at which the disease advances is roughly proportional to the carrying capacity, hence proportional to the local risk of zoonotic infection. Some geographical barriers, generated for example by riparian reforesting, can generate positive ecological impacts and can impede the spread of BSF to humans.

## Introduction

Stochastic epidemic models have been used to guide control policies for tick-borne infectious diseases [1–3]. These models typically assume that vector and host populations are homogeneous, disregarding the movement of infected individuals and the consequent spatial spread of infectious diseases [4]. Nonetheless, reaction-diffusion equations can be used to incorporate the spatial movement of individuals into stochastic epidemic models and predict the spatial advance of a disease [5–14]. In this approach, individuals are divided into a set of subgroups, each of which has its own stochastic dynamics described by a differential equation system, and adjacent subgroups are coupled by individual random movements with constant velocity [15, 16].

A remarkable example of a spatial spread system dependent on amplifier hosts is the Brazilian Spotted Fever (BSF), a highly lethal zoonotic disease caused by the bacteria *Rickettsia rickettsii*, transmitted by the tick *Amblyomma sculptum* Berlese, 1888 [17], (*Amblyomma cajennense* complex) (Ixodida: Ixodidae), and whose basic reproduction number (*R*_0_ ≈ 1.7) was recently calculated through a next-generation matrix approach [18]. Specifically, in the transmission of this disease, the vector *A. sculptum* is unable to maintain the *R. rickettsii* transmission cycle by transovarial transmission so that amplification by a reservoir host is required [19]. In Brazil, the maintenance of *R. rickettsii* depends primarily on the constant introduction of susceptible capybaras *Hydrochoerus hydrochaeris* [20, 21], which act as amplifiers and guarantee the constant creation of new cohorts of infected ticks [19, 22, 23]. Additionally, since ticks are limited in their mobility, *R. rickettsii* can spread over geographical areas by the movement of infected capybaras carrying either infected ticks from endemic areas or by transmitting the disease directly to susceptible ticks in neighboring regions. Currently, in agricultural endemic BSF areas, population densities of capybaras have reached numbers up to 40 times higher than those recorded in natural environments such as the Amazon and Pantanal [24] and thus, the risk of human infection has increased significantly over the last three decades [22, 25]. Notwithstanding the average abundance index of the groups of capybaras in southeastern Brazil has been reported in 50.55 individuals [26].

In southeastern Brazil, genetic analyses have confirmed a rapid spatial expansion of capybaras with evidence of secondary contacts between phylogroups [27]. In this region, the formation of capybaras subgroups and their migration occurs chiefly when they leave in search of food [27–29]. However, young capybaras can also migrate after the occurrence of agonistic behaviors [29, 30] and at the beginning of the sexual maturity [31]. The maximum and mean dispersal distances of capybaras have been reported in 5600 m and 3366 m, respectively [32, 33]. Moreover, it has been found that the home range of capybara groups differs in the different countries of South America. For instance, it covers from 6 to 16 ha in Venezuela [34], 11.3 to 27.6 ha in Argentina [35], 56 ha in Colombia [36] although up to 183 ha in Paraguay [37] or even from 196 ha [38] to 200 ha in Brazil [39].

The infection by *R. rickettsii* among different populations of capybaras and ticks in a homogeneous space was previously modeled [3]. In this preceding approach, two main risk factors for the *R. rickettsii* dissemination were identified: the current high birth rate of capybaras in endemic areas and the straightforward generation of new endemic areas due to the fact that a single infected capybara with just one infected tick attached is enough to trigger the disease in a non-endemic area. However, the risk of dissemination may be greater if it is considered: *i*) the current increase of the carrying capacity, determined by the abundance of sugarcane crops, the main food source of capybaras in São Paulo [40], *ii*) the ubiquitous distribution of the vector *A. sculptum* in the state of São Paulo [17, 41, 42] and *iii*) the large number of rivers in the region, through which capybaras can migrate [40].

This work aims to model a reaction-diffusion system that considers the spatial structure of capybaras to predict the spatial diffusion of the BSF in São Paulo and to assess potential preventive and control interventions. We calculate the BSF propagation and verify if the model describes the reported spatial-temporal spread of BSF. In addition, we create different scenarios to evaluate the effectiveness of preventing the capybaras’ exodus to control the spatial spread of the *R. rickettsii* and consequently prevent BSF human cases. This work contributes to the development of forthcoming mathematical and computational studies focused on the dynamics and prevention of vector-borne infectious diseases.

## Results and discussion

The main application of our reaction-diffusion system for the spread of the BSF is the design of control strategies to prevent or at least minimize the spread of this disease to humans. To address this problem, we verify if our model can describe the observed spatial-temporal spread of the BSF in the state of São Paulo by simulating a Markov stochastic process describing the *R. rickettsii* infection among *H. hydrochaeris* and *A. sculptum*.

**Fig 1:**
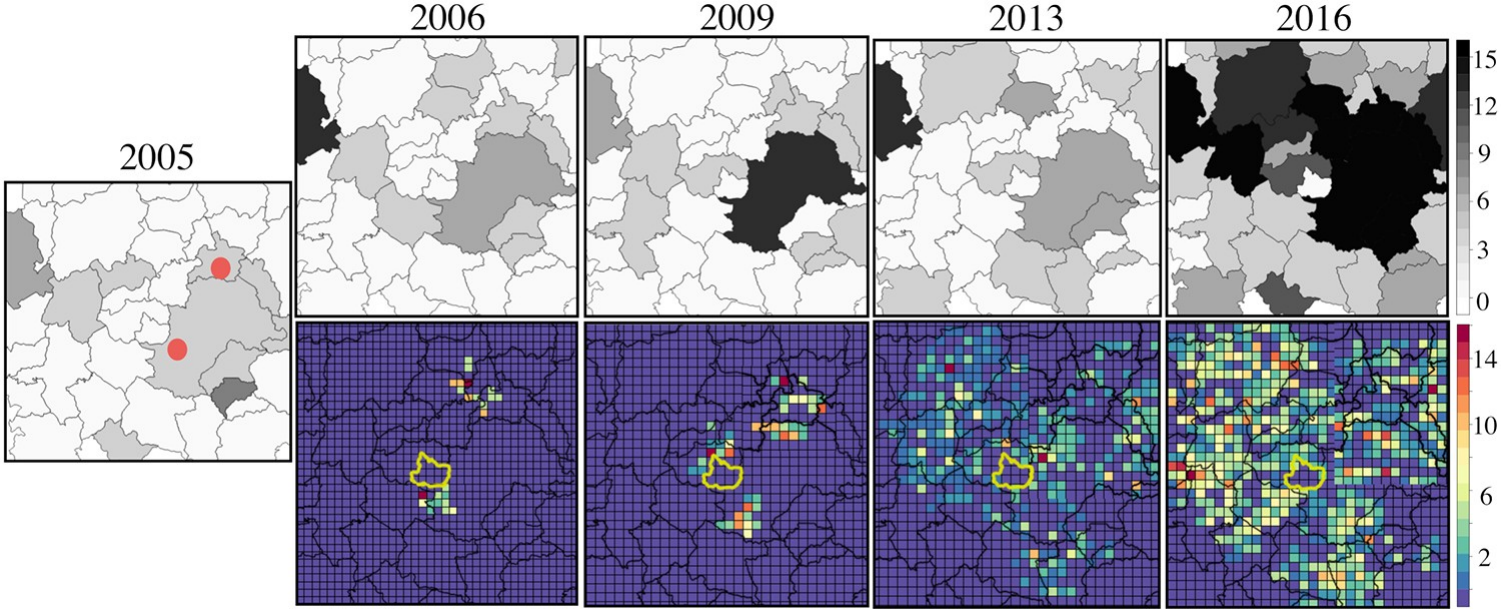
Comparison of the spread of human BSF cases with stochastic simulations from 2005 to 2016. Top: Spread of human BSF cases from 2005 to 2016. Red dots represent the two starting points of the simulations in which the disease has spread since 2005. North dot is located in Jaguariuna and south dot in Campinas. Bottom: Stochastic simulation results from 2005 to 2016 considering the distribution of sugarcane crops. Yellow polygon represents the municipality of Hortolândia, which reported no cases or sugarcane crops, nor migration of infected individuals in the proposed reaction-diffusion model.

Fig 1 shows a comparison of the spread of human BSF cases from 2005 to 2016 with the results of the stochastic simulations of the reaction-diffusion model considering the same time period. It can be noted that the stochastic simulations correspond with the spatial propagation of the observed cases of BSF in humans.

**Fig 2:**
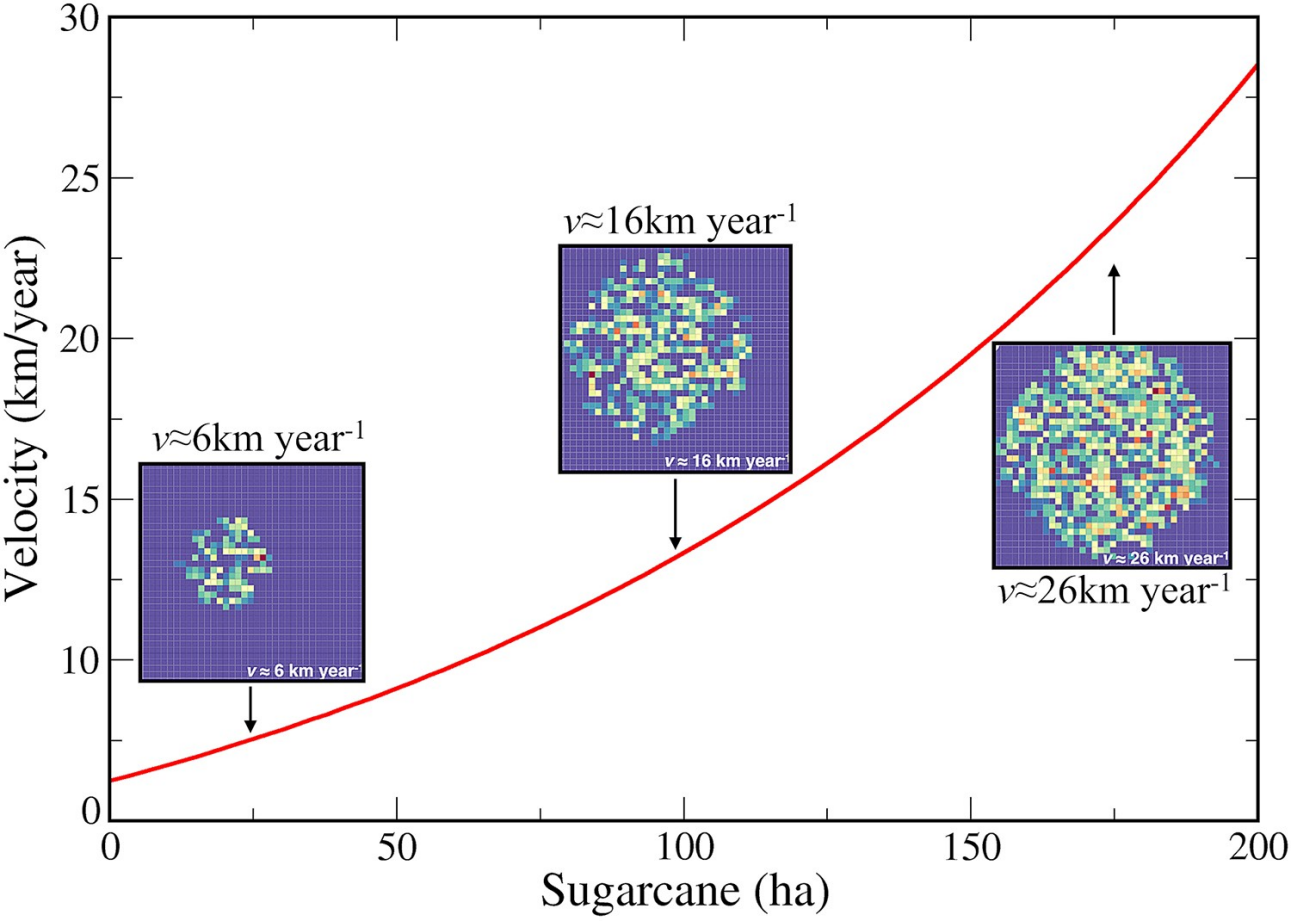
BSF propagation velocity and carrying capacity considering scenarios of a homogeneous amount of sugarcane. In these scenarios, the migration of infected individuals is symmetrical due to the homogeneous distribution of the sugarcane.

We found that migration and infections are null in areas without sugarcane, as in the central region of Hortolândia (Fig 1). In these sugarcane-free areas, no cases have been reported either, coinciding with the projections of our reaction-diffusion model. We also found that the propagation velocity increases as the carrying capacity becomes greater (Fig 2). A propagation velocity of BSF was calculated in 6 km yr^−1^ considering a homogeneous sugarcane of 10ha and in 10 km yr^−1^ considering a homogeneous amount of 59 ha, which is very close to the velocity of propagation obtained from Eq 8 (≈ 11.6 km/yr^−1^), considering *ϕ* = *μ*_*C*_ − *δ*_*C*_. A homogeneous amount of 100 ha generated a propagation velocity of 16 km yr^−1^ and a homogeneous quantity of 200 ha generates a propagation velocity of 26 km yr^−1^ as shown in Fig 2. In these scenarios, the migration of infected individuals is symmetrical due to the homogeneous distribution of the sugarcane.

**Fig 3:**
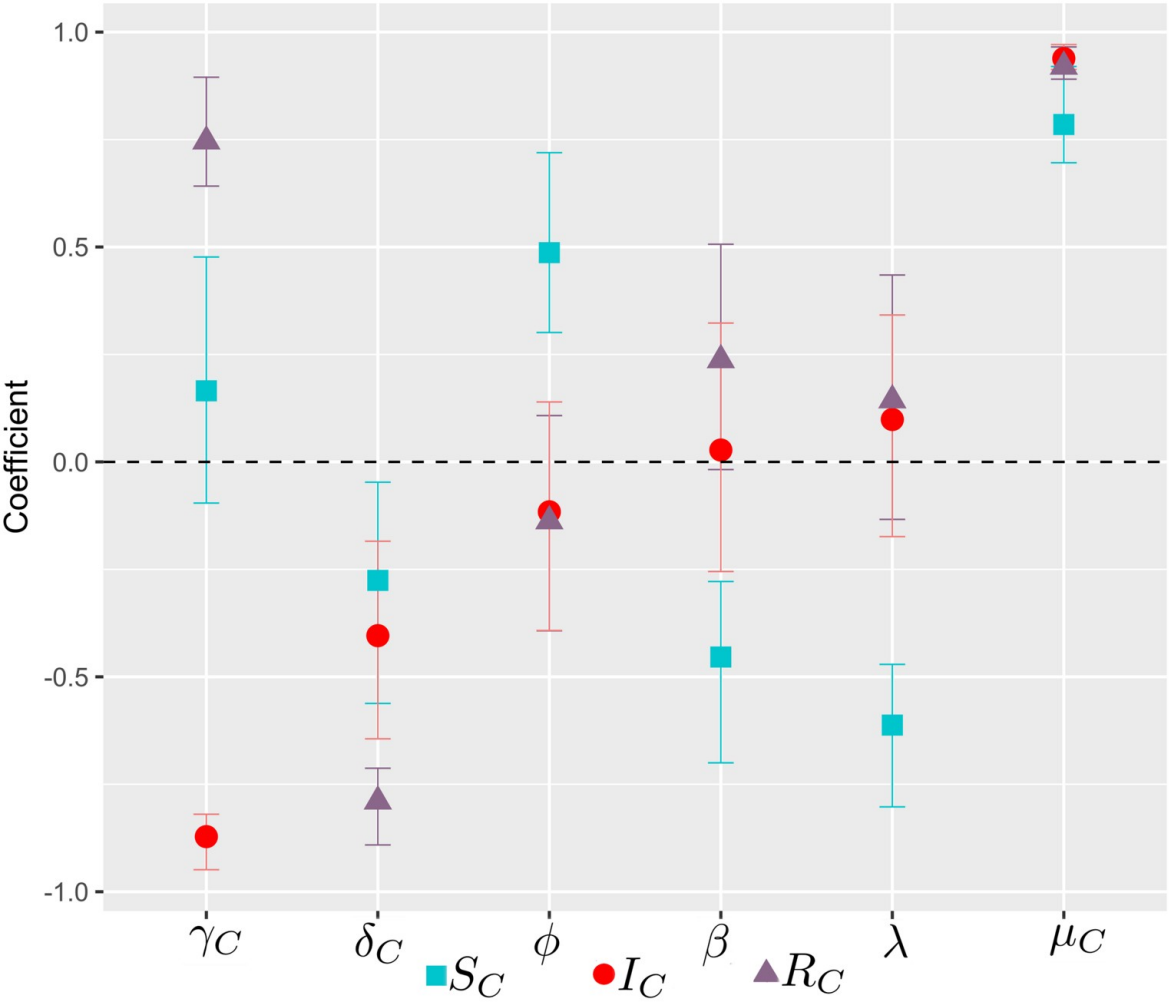
Partial rank correlation coefficient (PRCC) between each parameter and the average migratory population of capybaras. The birth rate of capybaras is the most influential parameter in affecting the number of migratory capybaras.

Sensitivity analysis shows that the uncertainties in estimating the values of the birth rate of capybaras are the most critical in affecting the prediction of the number of migratory susceptible, infected and recovered capybaras, as shown in Fig 3. In fact, in the specific case of infected capybaras, the unique factor that significantly and positively impacted their migration was their birth rate (PRCC = 0.94; 99% CI = 0.91 - 0.97). This positive value in the PRCC of the birth rate implies that when the value of this input variable increases, the future number of migratory capybaras will also increase. Furthermore, the future number of infected migratory capybaras decreases significantly as the recovery (PRCC = -0.87; 99% CI = -0.95 - -0.82) and death (PRCC = -0.41; 99% CI = -0.64 - -0.18) rates increase, as also shown in Fig 3. The migration rate of capybaras (*ϕ*) only impacts significantly the number of susceptible migratory capybaras (PRCC = 0.48; 99% CI = 0.28 - 0.73).

**Fig 4:**
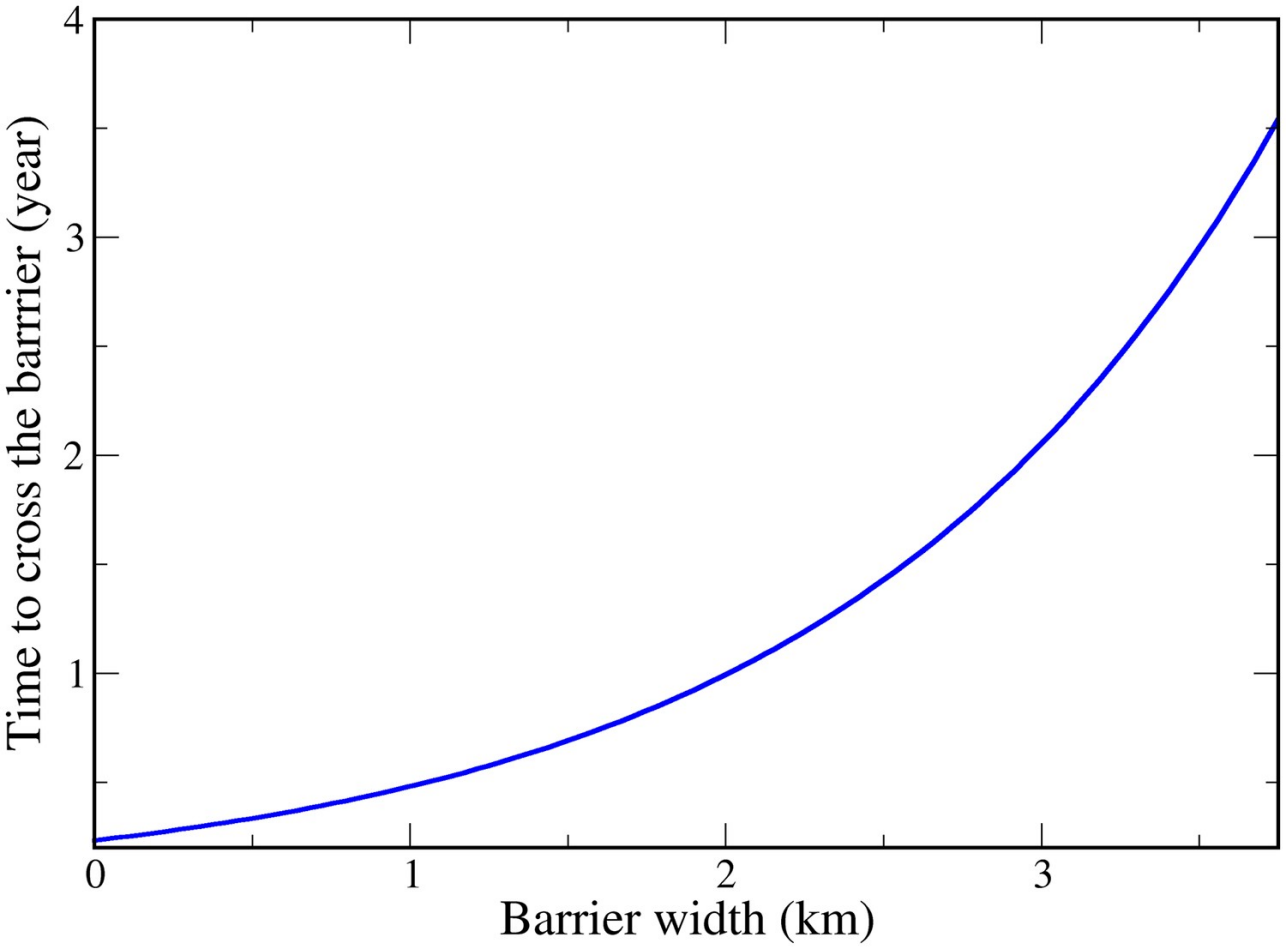
Relation of the barrier width and the time to cross the barrier. The disease is able to cross barriers of less than 2 km in the first year, barriers of 3km in the second year and barriers of 3.5 km in the third year.

In scenarios considering populations of ticks and capybaras surrounded by non-sugarcane barriers from 300 m to 4 km, regardless of the amount of cane where they were, the spatial movement of capybaras obeyed the distribution of the sugarcane. Migration of individuals was interrupted from a barrier width of 4km and therefore the spread of the disease was also intercepted. The disease was able to cross barriers of less than 2 km in the first year of simulation, barriers of 3km in the second year and barriers of 3.5 km in the third year. Fig 4 demonstrates the relation of the barrier width and the time to cross the barrier.

Since capybaras do not tend to move more than 500 meters from water bodies while foraging [43–45], riparian reforestation up to 500 meters around water resources could be an alternative to interrupting their access to sugarcane crops, reducing their supply of food, and consequently their birth rate. However, as found in this work, the distance of riparian reforestation should be greater since capybaras mean dispersal distances of 3366 m [32, 33] and home ranges up to 200 ha have been reported in South America [39]. Additionally, in areas with established groups of capybaras, riparian forested areas in the early stages are at risk in terms of plant survival due to the trampling of young plants of woody species [46]. Thus, riparian reforestation could be undertaken as a preventive strategy in areas where groups of capybaras have not yet established. Positively, riparian forests provide positive ecological impacts, such as biodiversity conservation, regularization of hydrological cycles, soil conservation, sediment retention, carbon fixation, and pollutant filtering [47–49].

Other strategies to reduce the birth rate of capybaras include the reduction of the carrying capacity, their removal, either by euthanasia or regulated hunting and their reproductive control [3]. Sugarcane crops are the main food source of capybaras in southeastern Brazil and the most important agricultural product in the region [40]. Furthermore, in this area, there is a constant availability of water sources, which allow the establishment of capybaras groups [40]. Certainly, controlling these two aspects is not feasible. Additionally, the removal or elimination of immune capybaras from endemic areas can generate a reintroduction of susceptible capybaras from uninfected areas [40], which could become infected given the long survival of unfed ticks in the pastures [50]. Reproductive control of capybaras was previously tested in Brazil [51]. However, considering that these animals escape quickly into the water when they feel in danger, the techniques of capture and containment of these animals for the performance of these surgical procedures should be better explored.

The inclusion of human/animals mobility models is a fundamental component in the analysis of the geographic spread of epidemics [52–54]. However, these models are of limited value when real mobility data is available [55, 56]. Since mobility data of capybaras is restricted, we assumed they move randomly in a local nonlinear infection dynamic yielding a reaction-diffusion model, as it can roughly report on the epidemic spread [57]. The usefulness of these models appears in data-scarce contexts, such as during infectious disease epidemics in low-income countries, when forecasting the best possible allocation of resources becomes necessary [55]. Indeed, these models lead to epidemic wavefronts which were observed, for example, in the spatial-temporal spread of the Black Death in Europe from 1347 to 1350 and that can predict spread patterns based solely on population size, population density, and travel distance [55, 58]. More sophisticated models constructed with a high degree of detail in which social, spatial and temporal heterogeneity are taken into account [59] can provide a more detailed understanding of the spread of BSF.

### Conclusion

We developed a reaction-diffusion system for the spread of an infectious disease by considering the spatial structure and migration of amplifier hosts. Our results indicate that as we vary the amount of food, the velocity at which the disease advances is roughly proportional to the carrying capacity, hence proportional to the local risk of zoonotic infection. Since our reaction-diffusion model considered a reasonably realistic spatial structure of capybaras and ticks and allowed to represent accurately the spatial dynamics of the Brazilian Spotted Fever in the state of São Paulo, it can allow the formulation of public actions focused on the prevention of these diseases and potentially other vector-borne diseases. The results of the sensitivity analysis can be used to focus prevention strategies on the birth rate of capybaras, as this analysis identified that this parameter (do to their estimation uncertainty) is the most important in the prediction of infected migratory capybaras. Some geographical barriers, generated for example by riparian reforesting, can generate positive ecological impacts and can impede the spread of BSF to humans.

## Materials and methods

### Non-spatial transmission dynamics

**Fig 5:**
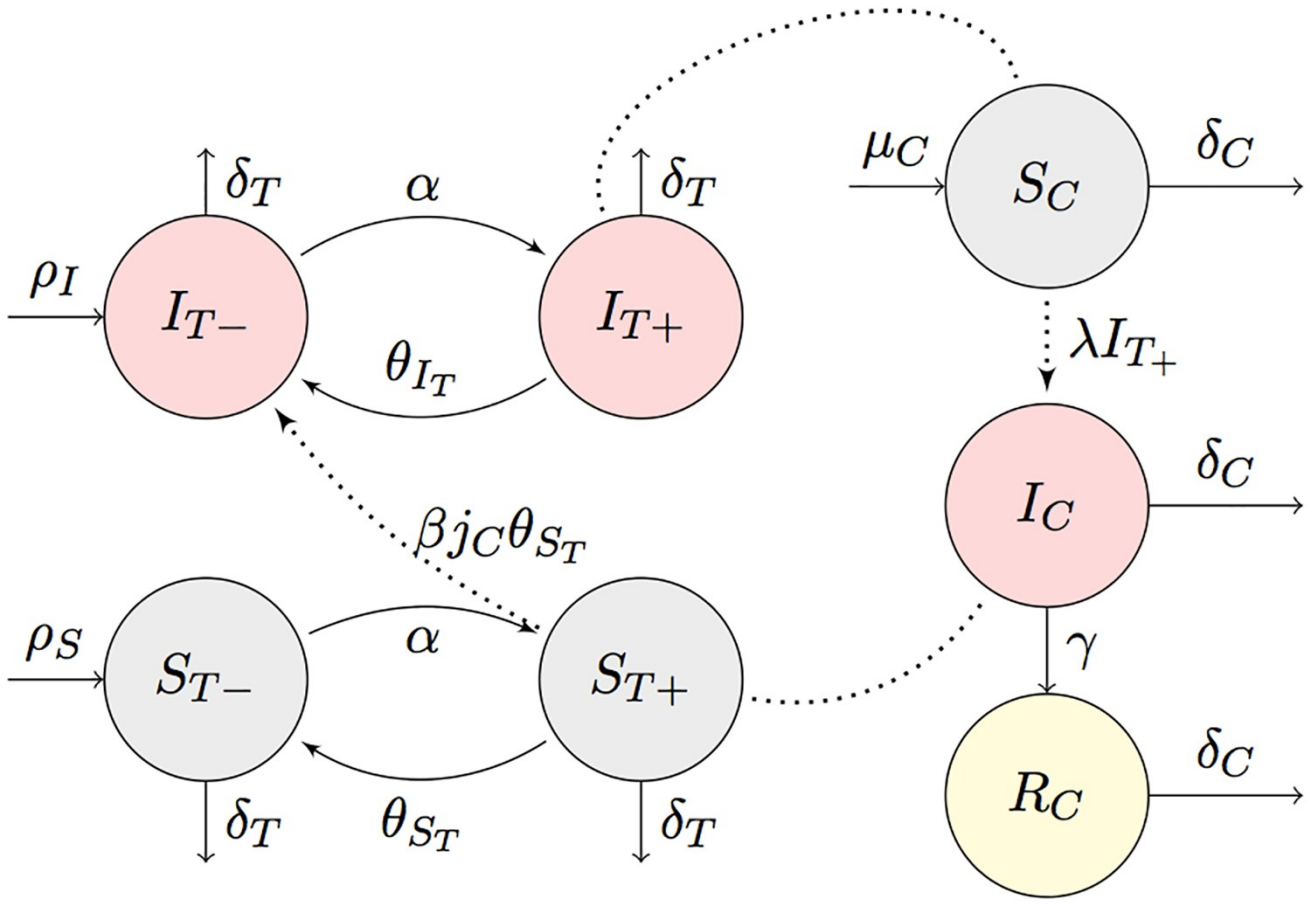
Schematic representation of the *R. rickettsii* transmission dynamics in populations of *H. hydrochaeris* and *A. sculptum* for each subgroup of capybaras and ticks.

Fig 5 schematically summarizes the BSF transmission dynamics for each subgroup of capybaras and ticks. Individuals are represented by *X*_*k*_, where *X* stands for the infectious state (susceptible *S*, infected *I*, and recovered *R*), *k* = *C* for capybaras or *k* = *T* for ticks. Ticks were classified according to their life cycle stages as larvae (L), nymphs (Y) and adults (A). Thus, *T* = *L*±, *Y*±, *A*±, where − represents detached from a capybara or + attached to it. In this way, the total capybara population is given by *N*_*C*_ = *S*_*C*_ + *I*_*C*_ + *R*_*C*_ and the total tick population by *N*_*T*_ = *S*_*L*−_ + *S*_*L*+_ + *S*_*Y*−_ + *S*_*Y*+_ + *S*_*A*+_ + *S*_*A*−_ + *I*_*L*−_ + *I*_*L*+_ + *I*_*Y*−_ + *I*_*Y*+_ + *I*_*A*+_ + *I*_*A*−_.

In order to consider the seasonal one-year generation pattern of the tick *A. sculptum*, the model was adjusted to a semi-discrete time dynamics [60]. We refer to a semi-discrete dynamics as the particular class of hybrid dynamical system that undergoes continuous dynamics in ordinary differential equations most of the time and experiences discrete dynamics at some time instants [61]. In our model, larvae exclusively quest and feed from April to July for 110 days, nymphs from July to October for 104 days and adults particularly quest, feed and reproduce from October to March for 151 days [3, 60]. Thus, within each tick season the transmission dynamics is continuous and between the seasons it is discrete. Dynamic quantities of the *R. rickettsii* ransmission stochastic system are presented in Table 1.

**Table 1:** Dynamic quantities of the *R. rickettsii* transmission stochastic system.

Absolute number	Fraction	Absolute number per patch	Fraction at patch	
*S*_*C*_	*s*_*C*_ = *S*_*C*_/*N*_*C*_	*S*_*C*_(**r**, *t*)	*s*_*C*_(**r**, *t*)	Susceptible capybaras
*I*_*C*_	*j*_*C*_ = *I*_*C*_/*N*_*C*_	*I*_*C*_(**r**, *t*)	*j*_*C*_(**r**, *t*)	Infected capybaras
*R*_*C*_	*r*_*C*_ = *R*_*C*_/*N*_*C*_	*R*_*C*_(**r**, *t*)	*r*_*C*_(**r**, *t*)	Recovered capybaras
*S*_*L*+_	*s*_*L*+_ = *S*_*L*+_/*N*_*T*_	*S*_*L*+_(**r**, *t*)	*s*_*L*+_(**r**, *t*)	Susceptible attached larvae
*I*_*L*+_	*j*_*L*+_ = *I*_*L*+_/*N*_*T*_	*I*_*L*+_(**r**, *t*)	*j*_*L*+_(**r**, *t*)	Infected attached larvae
*S*_*Y*+_	*s*_*Y*+_ = *S*_*Y*+_/*N*_*T*_	*S*_*Y*+_(**r**, *t*)	*s*_*Y*+_(**r**, *t*)	Susceptible attached nymphs
*I*_*Y*+_	*j*_*Y*+_ = *I*_*Y*+_/*N*_*T*_	*I*_*Y*+_(**r**, *t*)	*j*_*Y*+_(**r**, *t*)	Infected attached nymphs
*S*_*A*+_	*s*_*A*+_ = *S*_*A*+_/*N*_*T*_	*S*_*A*+_(**r**, *t*)	*s*_*A*+_(**r**, *t*)	Susceptible attached adults
*I*_*A*+_	*j*_*A*+_ = *I*_*A*+_/*N*_*T*_	*I*_*A*+_(**r**, *t*)	*j*_*A*+_(**r**, *t*)	Infected attached adults
*S*_*L*−_	*s*_*L*−_ = *S*_*L*−_/*N*_*T*_	*S*_*L*−_(**r**, *t*)	*s*_*L*−_(**r**, *t*)	Susceptible detached larvae
*I*_*L*−_	*j*_*L*−_ = *I*_*L*−_/*N*_*T*_	*I*_*L*−_(**r**, *t*)	*j*_*L*−_(**r**, *t*)	Infected detached larvae
*S*_*Y*−_	*s*_*Y*−_ = *S*_*Y*−_/*N*_*T*_	*S*_*Y*−_(**r**, *t*)	*s*_*Y*−_(**r**, *t*)	Susceptible detached nymphs
*I*_*Y*−_	*j*_*Y*−_ = *I*_*Y*−_/*N*_*T*_	*I*_*Y*−_(**r**, *t*)	*j*_*Y*−_(**r**, *t*)	Infected detached nymphs
*S*_*A*−_	*s*_*A*−_ = *S*_*A*−_/*N*_*T*_	*S*_*A*−_(**r**, *t*)	*s*_*A*−_(**r**, *t*)	Susceptible detached adults
*I*_*A*−_	*j*_*A*−_ = *I*_*A*−_/*N*_*T*_	*I*_*A*−_(**r**, *t*)	*j*_*A*−_(**r**, *t*)	Infected detached adults

Susceptible capybaras *S*_*C*_ can be infected by an attached tick at a rate λ. All capybaras have the same susceptibility and there is no increased death rate *δ*_*C*_ of infected individuals due to disease. Once capybaras are infected, they keep the *R. rickettsii* in the bloodstream for 7 to 10 days [21], during which the infection of new susceptible ticks that feed on it can occur at rate *β*. After this period, capybaras recovered at a rate *γ* and become immune to the disease. As capybaras natality depends primarily on the availability of food sources, as is typically the case of rodents [62], in the proposed model the birth rate *μ*_*C*_ of the capybara population was determined by the amount of sugarcane in the region obeying the function:

$$\begin{array}{c} \mu_{C}=[\mu_{0}+\delta \mu (1-e^{-c\left(r\right)/\bar{c}})], \end{array}$$ (1)

where *μ*_0_ is the reproduction rate in the absence of sugarcane and *δμ* is the increase in birthrate to its maximum if the sugarcane concentration *c*(**r**) at location **r** exceeds the spatial mean $\bar{c}$. A birth rate close to zero was considered in areas without sugarcane, and a maximum birth rate, *μ*_*C*_ = 1/136 *d*^−1^ was considered in areas with a maximum amount of sugarcane, as described below. This value considers a maximum litter size of capybaras reported in 6.1 pups [63, 64].

As it is also shown in Fig 5, ticks can attach at a rate *α*, detach at a rate *θ*_*T*_ and die at a rate *δ*_*T*_. The production rate *ρ* of *N*_*T*_ is assumed to be proportional to the total number of susceptible and infected attached ticks of the previous generation. Infected adult ticks have a lower production rate *ρ*_*I*_ than susceptible adult ticks *ρ*_*S*_, and the fraction of offspring by infected ticks is given by *a*_*S*_ = 305/532 and *a*_*I*_ = 228/532. The definition of the rates involved in the non-spatial transmission dynamics is specified in Table 2. This system of reactions can also be described by a coupled differential equation system,

*For ticks:*

$$\begin{array}{cc} {\dot{S}}_{T_{-}} & =\rho_{S}S_{+}+\theta_{S_{T}}S_{T_{+}}-\alpha S_{T_{-}}-\delta_{T}S_{T_{-}},\\ {\dot{I}}_{T_{-}} & =\rho_{I}I_{+}+\theta_{I_{T}}I_{T_{+}}-\alpha I_{T_{-}}+\beta j_{C}\theta_{S_{T}}S_{T_{+}}-\delta_{T}I_{T_{-}},\\ {\dot{S}}_{T_{+}} & =\alpha S_{T_{-}}-\beta j_{C}\theta_{S_{T}}S_{T_{+}}-\theta_{S_{T}}S_{T_{+}}-\delta_{T}S_{T_{+}},\\ {\dot{I}}_{T_{+}} & =\alpha I_{T_{-}}-\theta_{I_{T}}I_{T_{+}}-\delta_{T}I_{T_{+}}, \end{array}$$ (2)

*For capybaras:*

$$\begin{array}{cc} {\dot{S}}_{C} & =\mu_{C}N_{C}-\lambda s_{C}I_{T_{+}}-\delta_{C}S_{C}\\ {\dot{I}}_{C} & =\lambda s_{C}I_{T_{+}}-\gamma I_{C}-\delta_{C}I_{C},\\ {\dot{R}}_{C} & =\gamma I_{C}-\delta_{C}R_{C}, \end{array}$$ (3)

which has been previously studied [3] not only for the stationary state but also on the effect of rates changes.

**Table 2:** Definition of the rates involved in the tick-capybara-disease stochastic system.

Rate	Definition	Unit rate	Value [Range]
*μ*_*C*_	Capybara birth rate	1/days	0.005 [24, 51] [0.0027,0.0081]
*δ*_*C*_	Capybara death rate	1/days	0.002 [24] [0.0019,0.0032]
*γ*_*C*_	Capybara recovery rate	1/days	0.027 [22] [0.0138,0.041]
*ρ*_*S*_, *a*_*S*_	Birthrate of susceptible parent ticks	1/yr	*ρ*_*S*_ = 2709; *a*_*S*_ = 305/352 [19]
*ρ*_*I*_, *a*_*I*_	Birthrate of infected parent ticks	1/yr	*ρ*_*I*_ = 532; *a*_*I*_ = 228/532 [19]
*β*_*L*_,_*Y*_	Transmission rate from capybara to larvae or nymphs	1/days	*β*_*L*_ = 0.0003 [19, 21], *β*_*Y*_ = 0.0007 [19, 21]
λ_*L*_,_*Y*_,_*A*_	Transmission rate from larvae, nymphs and adults to capybaras	1/days	λ_*L*_ = 0.00009 [21] [8.19 × 10^−7^, 1.36 × 10^−4^]; λ_*Y*_ = 0.046 [21][0.0224, 0.0661];λ_*A*_ = 0.046 [21] [0.0004,0.0683]
*α*_*L*_,_*Y*_,_*A*_	Attachment rates for the various types of ticks	1/days	*α*_*L*_ = 0.003 [19] [0.0016,0.0048];*α*_*Y*_ = 0.006 [19] [0.0033,0.0099]; *α*_*A*_ = 0.009 [19] [4.25 × 10^−5^, 0.0135]
*θ*_*L*_,_*Y*_	Detachment rate of larvae and nymphs	1/days	*θ*_*L*_ = 0.0009 [19], *θ*_*Y*_ = 0.0016 [19]

### Simulations

The proposed reaction-diffusion system was implemented in the R language using the Gillespie algorithm [65, 66]. All parameters were estimated using data generated from *ex situ* field works in southeastern Brazil. A full list of the model’s reactions and parameters used in the simulations is given in Table 1. Groups of capybaras comprise a maximum of 50 individuals [24, 26, 67, 68] in all simulations.

#### Target area

**Fig 6:**
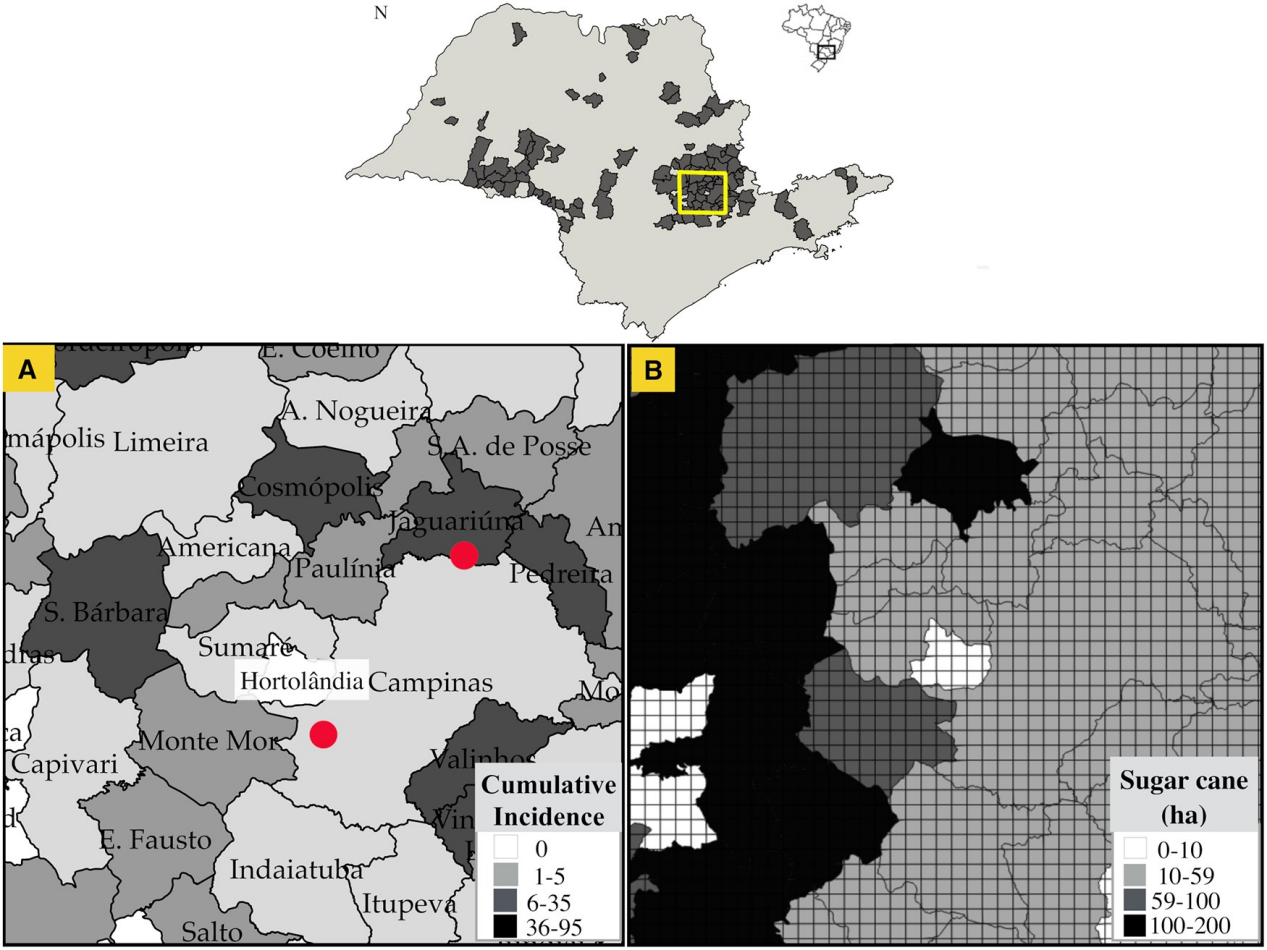
Target area. Yellow square represents the study area conformed by a grid of 50×50 pixels at regular intervals of 2 km in municipalities with human BSF at southeastern São Paulo. **A.** Spatial distribution of the cumulative incidence of BSF cases from 1985 to 2016. Red dots represent the two starting points of the simulations in which the disease has spread since 2005: Jaguariuna and Campinas **B.** Sugarcane distribution in the study area at the year 2016.

To showcase our approach, we considered a study area of 10 000 km^2^ at the southeastern state of São Paulo, which was divided into subregions of 4 km^2^ (area of a capybara subgroup). This division was included in the simulations by considering a grid of 50 × 50 pixels at regular intervals of 2 km, as shown in Fig 6. This area was selected because it has been identified as the most important area for the occurrence of human cases of BSF in the state of São Paulo [40]. In fact, this zone corresponds with three out of four spatial-temporal hotspot risk areas previously found through a retrospective space-time analysis [40]. Additionally, this area currently experiences an increment of the availability of sugarcane crops, which increases the carrying capacity of the region [40], the vector *A. sculptum* is ubiquitous [17, 41, 42, 69] and there is a constant availability of water sources, which generates a propitious environment for the establishment of capybaras groups, their ticks and consequently for *R. rickettsii*.

Thus, to verify if our model describes the observed spatial-temporal spread of the BSF in the state of São Paulo, we obtained the information of the annual human cases of each municipality of the state of São Paulo (S1 Data) from the São Paulo State Center of Epidemiological Surveillance (CVE/SES-SP) [70] and considered the annual Euclidean distance traveled by this disease from 1985 to 2016. We considered only BSF human cases from areas of transmission by *A. sculptum*, as previously determined [69]. Hence, we excluded BSF cases from the metropolitan area of São Paulo and from the São Paulo coast, where the implicated vectors have completely different ecological traits, in which capybaras play no role. After reports of the disease between 1920 and 1940, in which the lethality reached 80% in the states of São Paulo and Minas Gerais, BSF only re-emerged in 1985 in the municipality of Pedreira. In 1986, BSF was reported in Jaguariuna, traveling a distance of 15.4 km yr^−1^. Although the disease spread again in São Paulo at this time, detection and reporting of the disease began to be effective years later [71]. The Brazilian Information System for Notifiable Diseases (SINAN) was created in 1993 and it was not until then that new cases were reported again in Jaguariuna. From 1993 to 1995 the disease reached the municipality of Campinas (10.8 km yr^−1^) and in 1996 reached Limeira (18.5 km yr^−1^). Considering only the largest distances of each year, from Campinas, the disease reached Monte Alegre do Sul in 1997 (23.1 km yr^−1^), Santo Antônio da Posse in 1998 (10.94 km yr^−1^) and Piracicaba in 2002 (11.9 km yr^−1^). After 8 years, in 2003, it reached the region of Ipeuná located at 90.40 km (11.3 km yr^−1^) and Oriente (43.55 km yr^−1^). In 2004, it reached the northwestern region of the state in Rio Preto (22.7 km yr^−1^) and after 10 years in 2005, it reached the western region in Marília (33.5 km yr^−1^) and the northern in Mococa (15.92 km yr^−1^). In 2007, it reached Cândido Mota (31.25 km yr^−1^) and Cruzalia (34.25 km yr^−1^), and in 2008 Maracaí (31.67 km yr^−1^). In 2009 the disease reached the northern border in Guairá (22.84 km yr ^−1^), in 2010 the eastern border in Silveiras (16.34 km yr ^−1^) and in 2011 the western border of the state (31.15 km yr ^−1^). In 2012 human cases occurred in Rancharia (25.7 km yr ^−1^) in 2013 in Iepê (24.8 km yr ^−1^) and in 2014 traveled the longest distance to the municipality of Fernandôpolis at 461.9 km (24.31 km yr ^−1^). Fig 6A shows the spatial distribution of the cumulative incidence of human cases of BSF in the study area from 1985 to 2016.

We obtained the annual geographical pattern of the sugarcane from 2005 to 2015 for each municipality from the Canasat-Area Project of the Brazilian National Institute for Space Research, which maps the sugarcane distribution of the state of São Paulo once a year using remote sensing imagery by the Landsat, CBERS and Resourcesat-I satellites with a spatial resolution of 30m, 20m and 23.5m, respectively [72]. Subsequently, we determined the average of sugarcane coverage of each municipality for each pixel by taking the total amount (ha) of sugarcane divided by the total number of pixels in a given municipality. For instance, in Fig 6B, it is shown the sugarcane amount (ha) of 2015 in the study area. We found that in São Paulo, the current average of sugarcane coverage in a pixel of 4km^2^ is about 59 ha and the maximum average of sugarcane coverage is 200 ha. We also considered that each initially established subgroup should be localized in a spatial region with sugarcane. Accordingly, we also consider susceptible capybaras subgroups (*N*_*C*_ = 50) around each initial central area.

#### Spatial spread

As capybaras are territorial animals typically distributed in groups in delimited areas [34–39], we considered capybaras subgroups at regular intervals *l* of 2 km, at grid locations **r** = (*r*_*x*_, *r*_*y*_). As in the non-spatial dynamics, capybaras and ticks have the same classification and stages. The dispersal dynamics is governed by a Markov process,

$$\begin{array}{c} X_{k}\left(r\right)\begin{array}{c} \phi (r^{\prime }|r)\\ ⇌\\ \phi (r|r^{\prime }) \end{array}X_{k}\left(r^{\prime }\right), \end{array}$$ (4)

where individuals of type *k* have a unique mobility rate *ϕ* that determines their travel between locations **r** and **r**′ which are vertices of a 2d square lattice *r*_*nm*_ = (*nl*, *ml*).

This allows generalizing the non-spatial coupled differential equation system describing the *R. rickettsii* dynamics,

$$\begin{array}{cc} \partial_{t}s_{C}\left(r,t\right) & =\mu_{C}N_{C}\left(r,t\right)-\lambda s_{C}\left(r,t\right)I_{T_{+}}\left(r,t\right)-\delta_{C}s_{C}\left(r,t\right)\\ & +\sum_{r^{\prime }}\left(\phi \left(r|r^{\prime }\right)s_{C}\left(r^{\prime },t\right)-\phi \left(r^{\prime }|r\right)s_{C}\left(r,t\right)\right),\\ \partial_{t}j_{C}\left(r,t\right) & =\lambda s_{C}\left(r,t\right)I_{T+}\left(r,t\right)-\gamma j_{C}\left(r,t\right)-\delta_{C}j_{C}\left(r,t\right)\\ & +\sum_{r^{\prime }}\left(\phi \left(r|r^{\prime }\right)j_{C}\left(r^{\prime },t\right)-\phi \left(r^{\prime }|r\right)j_{C}\left(r,t\right)\right),\\ \partial_{t}r_{C}\left(r,t\right) & =\gamma j_{C}\left(r,t\right)-\delta_{C}r_{C}\left(r,t\right)+\sum_{r^{\prime }}\left(\phi \left(r^{\prime }|r\right)r_{C}\left(r^{\prime },t\right)-\phi \left(r^{\prime }|r\right)r_{C}\left(r,t\right)\right), \end{array}$$ (5)

where *s*_*C*_(**r**, *t*), *j*_*C*_(**r**, *t*) and *r*_*C*_(**r**, *t*) are the fraction of susceptible, infected and recovered capybaras at patch **r**. *N*_*C*_(**r**, *t*) is the total number of capybaras at **r**, given by *N*_*C*_(**r**, *t*) = ∑_**r**_ = *S*_*C*_(**r**, *t*) + *I*_*C*_(**r**, *t*) + *R*_*C*_(**r**, *t*). Here, the dynamical equations for ticks are not represented, since we assume that susceptible and infected attached ticks are carried by capybaras and are diffused in this way. The tight connection of our discrete model to spatially continuous reaction-diffusion systems is trivial for our case, in which the travel rates associated with the mobility between neighboring subgroups occur in a grid. Thus, the general dispersal can be written as:

$$\begin{array}{c} \partial_{t}u\left(r,t\right)=\sum_{r^{\prime }}\left[\phi \left(r|r^{\prime }\right)u\left(r^{\prime },t\right)-\phi \left(r^{\prime }|r\right)u\left(r,t\right)\right], \end{array}$$ (6)

and specifically the dispersal to the neighboring sites as:

$$\begin{array}{c} \partial_{t}u\left(r,t\right)=\sum_{r^{\prime }\in U\left(r\right)}\left[\phi \left(r|r^{\prime }\right)u\left(r^{\prime },t\right)-\phi \left(r^{\prime }|r\right)u\left(r,t\right)\right], \end{array}$$ (7)

where *U*(**r**) are the four sites **r** ± (1, 0)*l* and **r** ± (0, 1)*l*, and *u*(**r**, *t*) is the place holder for one of the capybara compartments.

For sufficiently localized initial conditions, these systems can exhibit traveling waves with a constant velocity *v* [57]:

$$\begin{array}{c} v=2\sqrt{\lambda D(1-\gamma /\lambda )}\;∼\sqrt{\phi }, \end{array}$$ (8)

in which *D* = *l*^2^*ϕ*. Considering annual changes in the carrying capacity, it is expected that this velocity will not remain constant since it can not be assumed that susceptible, infectious, and recovered capybaras diffuse at an equal rate. Indeed, a spatial-temporal relationship between the occurrence of human rickettsiosis and sugarcane crops increment was verified by satellite hyperspectral imagery in São Paulo [40]. For this reason, we considered that the movement of capybaras depends on the spatial distribution and amount of sugarcane crops, as it is their main food source in this region. In this way, the probability associated with the migration site depended on the carrying capacity determined by the amount of sugarcane of the neighbors can be written as,

$$\begin{array}{c} \phi \left(r|r^{\prime }\right)=\phi_{max}-\left(\phi_{max}-\phi_{0}\right)e^{-c\left(r^{\prime }\right)/\bar{c}}, \end{array}$$ (9)

where *c*_**r**_ is the amount (ha) of sugarcane at location **r**. Thus, if there is no sugar cane the dispersal is *ϕ*_0_ and if there is a large amount of sugarcane it is *ϕ*_*max*_. Hence, an increment in the sugar cane density (carrying capacity) increases birth rates of capybaras, which in turn affect migration rates if the increment of capybaras population exceeds the carrying capacity of the region. This migration rate was adjusted in order to reproduce the observed spread of the disease from two starting points of the simulations or initial endemic central areas with growing capybaras populations, in which public health entities reported that the disease has spread since 2005: Jaguariuna and Campinas (Fig 6A) [70]. In these areas, the number of individuals considered corresponds with previous results obtained for endemic areas [3]: *S*_*C*_ = 5, *I*_*C*_ = 10, *R*_*C*_ = 35, *S*_*A*+_ = 1000 and *I*_*A*+_ = 5. Using the found migration rate, we determined the impact of the quantity of sugarcane on the propagation velocity of the BSF by considering four different scenarios with homogeneous sugar cane amount: 10 ha, 59 ha, 100 ha and 200 ha.

**Fig 7:**
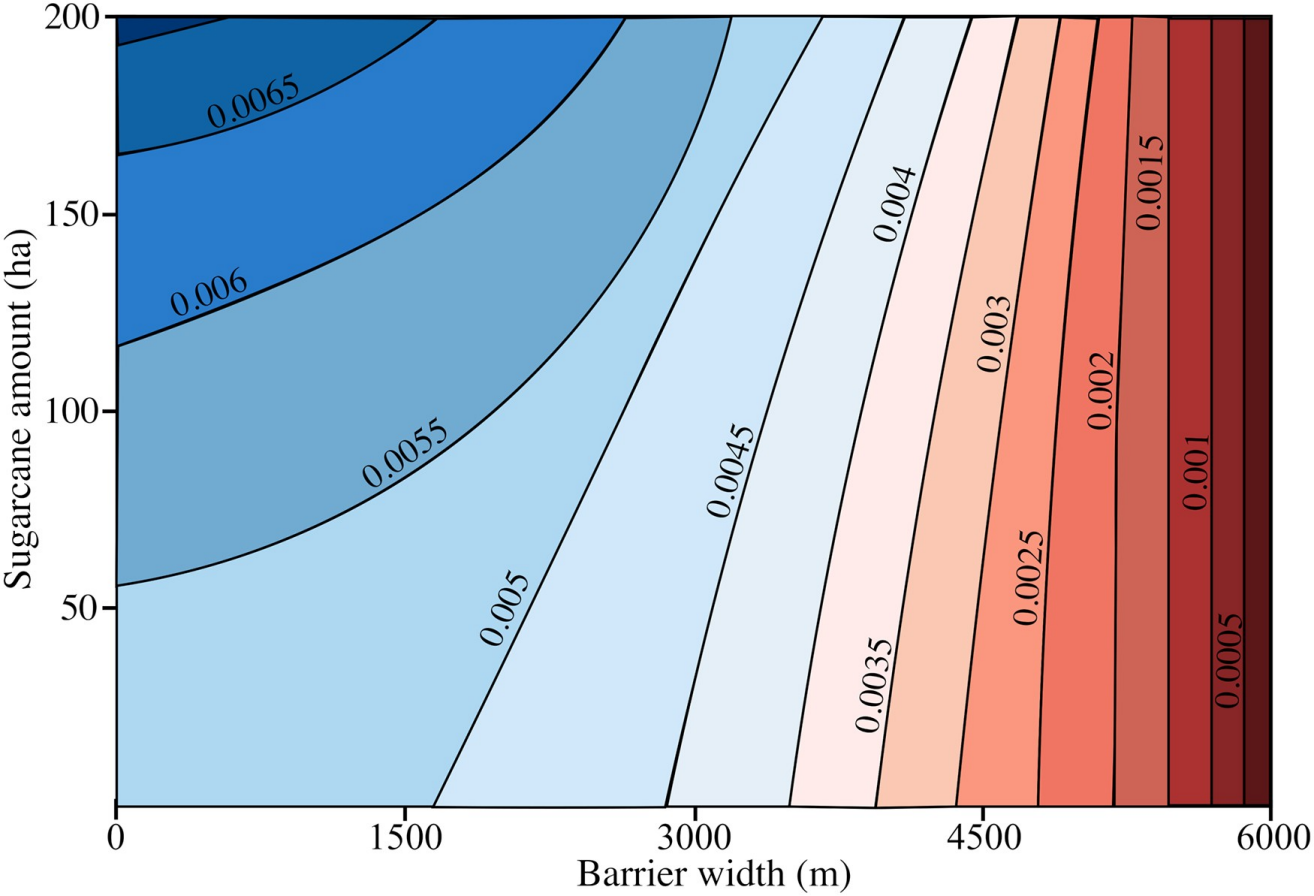
Migration *ϕ* rate depending on the sugarcane amount and the barriers width. The migration rate is higher as there is a greater amount of sugarcane and a smaller width of the barriers.

Furthermore, as capybaras natality depends primarily on the availability of food sources (Eq 1), we hypothesized that riparian (foodless areas) barriers might work as a strategy to block the access of capybaras to food sources (sugarcane crops), thereby decreasing their birth rate, and consequently preventing the spatial propagation and transmission of BSF to humans. Hence, two factors were determined, the amount of sugarcane and the width of these barriers. We considered a factor:

$$\begin{array}{c} \exp(-b(r)/\Delta ), \end{array}$$ (10)

which modifies the transition rate of Eq 9

$$\begin{array}{c} \phi \left(r|r^{\prime }\right)=[\phi_{max}-\left(\phi_{max}-\phi_{0}\right)e^{-c\left(r^{\prime }\right)/\bar{c}}]\exp\left(-b\left(r\right)/\Delta \right), \end{array}$$ (11)

in which we assumed that *ϕ*(**r**|**r**′) is also a function of the width of the barrier as shown in Fig 7, and *b*(*r*) increases with this width. Thus, if the barrier is much larger than the scale factor Δ the rate *ϕ* of going from *r* to *r*′ becomes zero, whereas if *b*(*r*) = 0, no barrier exists and the rate is not decreased. There are no data on the migratory behavior of capybaras in foodless regions, which means that the maximum distance that these amplifier hosts can migrate in regions deprived of food is unknown. Therefore, we simulated different scenarios considering natural barriers with different widths (from 300 m to 4 km) and three different maximum migration distances (2km, 4km, and 6km) [32–34]. This allows us to estimate the critical distance that a barrier must have in order to avoid the migration of infected individuals.

#### Uncertainty and sensitivity analysis

To quantify the impact of the parameters variation *α*, *μ*_*C*_, λ, *γ*, *δ*_*C*_ and *ϕ* on the abundance of susceptible, infected and recovered migratory capybaras and infected nymphs derived from the reaction-diffusion process, we combined uncertainty through the Latin hypercube sampling (LHS) with the robust Partial rank correlation coefficient (PRCC) method [73, 74]. The LHS procedure was implemented by dividing the range of values for a given parameter into equally one hundred intervals. As parameters ranges are unreported, the LHS was sorted from a set of uniform distributions [74] as shown in Table 2. Starting from this, model outputs were obtained of all possible combinations of parameters and the parameter and output values were transformed into their ranks. PRCC were calculated between each of the input variables (*α*, *μ*_*C*_, λ, *γ*_*C*_, *δ*_*C*_, *ϕ*) and the amount of susceptible, infected and recovered migratory capybaras.

## Supporting information

S1 Data Annual human cases of Brazilian Spotted Fever. (CSV)
